# MiR-429 suppresses the progression and metastasis of osteosarcoma by targeting ZEB1

**DOI:** 10.17179/excli2017-258

**Published:** 2017-05-05

**Authors:** Yi Deng, Fujun Luan, Li Zeng, Yanjun Zhang, Kunlong Ma

**Affiliations:** 1 Department of Oncology, Yongchuan Hospital of Chongqing Medical University, Chongqing 402160, China; 2 Department of Orthopedics, Yongchuan Hospital of Chongqing Medical University, Chongqing 402160, China; 3 Department of General Surgery, Yongchuan Hospital of Chongqing Medical University, Chongqing 402160, China

**Keywords:** miR-429, osteosarcoma, progression, EMT, ZEB1

## Abstract

MiR-429 functions as a tumor suppressor and has been observed in multiple types of cancer, but the effects and mechanisms of miR-429 in osteosarcoma are poorly understood. This study is performed to evaluate the functions of miR-429 in the progression of osteosarcoma. Firstly, the miR-429 expression in osteosarcoma tissues and osteosarcoma cells was detected using real time PCR, and the relationship between miR-429 expression and overall survival of osteosarcoma was analyzed. Secondly, the effects of miR-429 on the migration, invasion, proliferation and apoptosis of osteosarcoma cells were evaluated using transwell assay, wound-healing assay, CCK-8 assay and flow cytometry, respectively. Proteins related to epithelial-mesenchymal transition (EMT), E-cadherin, Vimentin, N-cadherin and Snail, were also detected using Western blot. Finally, the target gene of miR-429 in osteosarcoma was predicted and verified using dual luciferase assay and the expression correlation between them was analyzed using Pearson's correlation. MiR-429 was down-regulated in osteosarcoma tissues and osteosarcoma cells; the expression level of miR-429 was associated with the prognosis of osteosarcoma. High level of miR-429 in osteosarcoma cells significantly suppressed the migration, invasion and proliferation of cells but induced cells apoptosis. Furthermore, high level of miR-429 in osteosarcoma cells obviously increased the expression of E-cadherin protein but decreased the expression of Vimentin, N-Cadherin and Snail proteins. EMT inducer ZEB1 was the target gene of miR-429 and the expression of ZEB1 was negatively related to the miR-429 expression in osteosarcoma. In conclusion, miR-429 may functions as a tumor suppressor and be down-regulated in osteosarcoma. MiR-429 may suppress the progression and metastasis of osteosarcoma by down-regulating the ZEB1 expression.

## Introduction

MicroRNA, a small non-coding RNA, its differential expression is related to time phase, cell type and tissue (Lagos-Quintana et al., 2002[9]; Wienholds et al., 2005[25]). MiRNAs can regulate gene expression at a posttranscriptional level via base-pairing with mRNAs of target genes, a certain miRNA may regulate different genes' expression and a certain gene mRNA may be regulated by multiple miRNAs (Friedman et al., 2009[5]; Krek et al., 2005[7]). The target genes express proteins involved in different biological processes of cell functions, so miRNAs play multiple roles in many biological processes and animal development (Brennecke et al., 2003[1]; Chen et al., 2004[3]; Cuellar and McManus, 2005[4]). Similarly, the development of disease is also associated with aberrant miRNA expression in organism, like inherited diseases (Mencia et al., 2009[14]), heart disease (Thum et al., 2007[21]). In addition, plenty of miRNAs also have been determined to link with cancer. Over-expression of miR-155 down-regulates DNA mismatch repair protein MLH1 and may cause colon cancer (Valeri et al., 2010[22]). Deficiency of miR-324a may be related to the poor survival of NSCLC (Vosa et al., 2011[23]). Down-regulation of miR-30a is associated with epithelial-to mesenchymal transition (EMT) in non-small cell lung cancer (Kumarswamy et al., 2012[8]).

Accumulating researches have been determined to focus on the roles of miR-429 in tumorigenesis, which is a member of miR-200 family. Members of miR-200 family are down-regulated during EMT in normal murine mammary epithelial cells (NmuMG) (Korpal et al., 2008[6]). miR-429 expression is negatively correlated with the colorectal cancer progression and overexpression of miR-429 may suppress cell apoptosis, while the tumor inhibiting effect of miR-429 in colorectal carcinoma may function via targeting Onecut2 (Li et al., 2013[12]; Sun et al., 2014[20]). miR-429 functions as a tumor suppressor by down-regulating the expression of transcriptional repressor ZEB1 in oral squamous cell carcinoma and breast cancer (Lei et al., 2015[11]; Ye et al., 2015[26]), while suppresses invasion and promotes apoptosis through targeting Bcl-2 and SP1 in esophageal carcinoma (Wang et al., 2013[24]). These studies suggest that miR-429 may play important roles in multiple tumor diseases via regulating different target genes' expression.

Osteosarcoma (OS) is one of the most common primary tumors, and commonly formed in the bones of arms and legs of children and adolescents. OS is apt to metastasis and recurrence with low survival rate (Ottaviani and Jaffe, 2009[15]). But there is few evidence to determine the functions of miR-429 in OS progression (Liu et al., 2014[13]). To verify the effects of miR-429 in the tumorigenesis of OS, the expression level of miR-429 in OS tissues and OS cell lines was determined, furthermore, the effects of miR-429 overexpression on OS cells' invasion, metastasis, proliferation, apoptosis and EMT were evaluated. In addition, target gene predication and verification of miR-429 and the correlation analysis were also performed.

## Material and Methods

### Clinical specimens

A total of 50 OS tissue and 20 non-cancerous bone tissues were obtained from 50 patients with osteosarcoma who underwent surgery at Yongchuan Hospital of Chongqing Medical University from June 2008 to July 2011. Those patients who received chemo- or radiotherapy before surgery were excluded and OS tissues were confirmed using pathological method. All patients were followed up regularly after surgery until January 2014 (5 years) or until death. This study has been approved by the Ethics Committee of Yongchuan Hospital of Chongqing Medical University (Chongqing, China) and each patient has given the written informed consent.

### Cell culture and transfection

Human fetal osteoblastic cell line 1.19 (hFOB 1.19) and OS cell lines (Saos2 and U2OS) were purchased from ATCC (American Type Culture Colleciton) and cultured in DMEM (Gibco, Los Angeles, USA) supplemented with 10 % FBS (Hy-Clone, Logan, USA) at 37 ºC in a humidified atmosphere with 5 % CO_2_. HEK-293T cells were cultured in DMEM supplemented with 10 % FBS and prepared for dual luciferase assay. Saos2 cells and U2OS cells were transfected with mimics NC or miR-429 mimics according to the instruction of Lipofectamine 2000 (Invitrogen, Carlsbad, USA) respectively, and EMT markers were detected using Western blot 48 h post-transfection. Furthermore, Saos2 cells and U2OS cells were transfected with mimics NC or miR-429 mimics or inhibitor NC or miR-429 inhibitor respectively for expression detection of ZEB1 mRNA and protein. Mimics NC, miR-429 mimics, inhibitor NC and miR-429 inhibitor were purchased from GenePharma (Shanghai, China).

### Transwell invasion assay

24-well Transwell insert chambers (8-μm pore size, Corning, USA) pretreated with Matrigel (BD Biosciences) were used for invasion assay. 4 ×10^4^ cells (Saos2 or U2OS) transfected with mimics NC or miR-429 mimics were seeded in each upper well with free-serum medium, and the lower chamber well was filled with DMEM supplemented with 10 % FBS. After 24 h incubation at 37 ºC in a humidified atmosphere with 5 % CO_2_, cells invaded through the Matrigel to the lower surface of filter were fixed with 4 % paraformaldehyde followed by staining with 0.1 % crystal violet and imaged under microscope. The experiments were performed three times independently.

### Wound-healing assay

Cellular migration ability was determined using wound-healing assay. Mimics NC- or miR-429 mimics-transfected OS cells (Saos2 or U2OS) were seeded into each well of 12-well plates and made a straight line wound with a pipette tip when cell layers reached confluence. Then cells were washed with PBS and cultured in serum-free medium for 24 h. The closure of the wounds in each well was evaluated using an inverted microscopy. The experiments were performed three times independently.

### Cell viability assay

CCK8 assay was used to evaluate cell viability. Saos2 or U2OS cells (5×10^3^ cells) transfected with mimics NC or miR-429 mimics were seeded in each well of 96-well plates. CCK8 solution (10 μl) (Beyotime, Jiangsu, China) was added to each well after incubation for 24 h, 48 h and 72 h, respectively, and further incubated for 1 h. Absorbance of each well at 450 nm was measured on a microplate reader. The experiments were performed three times independently.

### Cell apoptosis assay

Annexin V-FITC Apoptosis Detection Kit (Beyotime) and flow cytometry were used to determine cell apoptosis. 5×10^4^ cells were suspended with 195 μl Annexin V-FITC binding buffer after 48 h transfection followed by adding 5 μl Annexin V-FITC and 10 μl PI staining solution and further incubated for 10 min at room temperature in the dark. Cell apoptosis was detected using flow cytometry with CellQuest software. The experiments were performed three times independently.

### Dual luciferase assays

The pmirGLO vector was purchased from Promega (Madison, USA) and used for dual luciferase assay. The target sequence of miR-429 in the 3'-UTR region of ZEB1 was predicted using Targetscan software. 3'-UTR sequence of ZEB1 mRNA including predicted miR-429 binding site and 3'-UTR sequence with corresponding mutation were ligated into the pmirGLO vector, respectively. And HEK-293T cells, seeded in a 24-well plate with 1×10^5^ cells per well, were cotransfected with 500 ng pmirGLO-ZEB1 3'-UTR wt or pmirGLO-ZEB1 3'-UTR mut and 15 pmol mimics NC or miR-429 mimics or inhibitor NC or miR-429 inhibitor using Lipofectamine 2000 (Invitrogen), respectively. Each group was treated with three duplications. After 48 h of incubation, the luciferase activities were detected by Dual Luciferase Reporter Assay System (Promega). 

### Quantitative real-time PCR

Total RNA was extracted from OS tissues or OS cells using Trizol reagent (Invitrogen) to analyze the miR-429 expression or ZEB1 mRNA expression. The miR-429 level was quantified by the TaqMan miRNA Assay (Life Technologies, Grand Island, USA) and 7900 Real-Time PCR System (Applied Biosystems, Foster City, USA) according to the instructions, U6 expression level was detected as internal normalized reference. The primers used for amplification of miR-429 and U6 were purchased from Applied Biosystems. The detection of ZEB1 mRNA level was performed using PrimeScript RT reagent Kit and SYBR Premix Ex Taq (Takara, Dalian, China), GAPDH mRNA level was detected as internal normalized reference. Primers for ZEB1 and GAPDH were as follow: ZEB1 primers (forward, 5′-TTAGTTGCTCCCTGTGCAGTT-3′ and reverse, 5′-TAGGAGCCAGAATGGGAAAAG-3′), GAPDH primers (forward, 5'-ACGGGAAGCTCACTGGCATGG-3' and reverse, 5'-GGTCCACCACCCTGTTGCTGTA-3').

### Western blot

Total protein was extracted from OS cells using RIPA buffer (Beyotime, Shanghai, China) and quantified using BCA assay kit (Bio-rad, China) as per the manufacture's instruction. Then total protein (40 μg) from each sample was separated using SDS-PAGE and transferred to a PVDF membrane. The membranes were probed with anti-E-cadherin antibody (Abcam, ab76055), anti-Vimentin antibody (Abcam, ab8978), anti-N-Cadherin antibody (Abcam, ab98952), anti-Snail antibody (Abcam, ab117866), anti-ZEB1 antibody (Abcam, ab181451) and anti-β-actin antibody (Abcam, ab8224) after blocked with 5 % non-fat milk. Then the membranes were further probed with HRP-conjugated second antibody (Abcam, ab6728). β-actin was used as an internal control and protein bland was detected with enhanced chemiluminescence (Amersham Pharmacia). Densitometry of Western blots was quantified using NIH ImageJ software (Bethesda, USA). Blotting images and densitometry results were representative from 3 repeats.

### Statistical analysis

Kaplan-Meier method was used to plot the survival curves based on miR-429 relative expression and overall survival, statistical significance was determined by the log-rank test. Continuous data were presented as mean ± SD and analyzed using GraphPad Prism 5 software, comparison between groups was performed by an independent *t* test, *P* value less than 0.05 was considered statistically significant. Correlation analysis between miR-429 relative expression and ZEB1 relative expression in OS tissues was performed using Pearson's correlation.

## Results

### MiR-429 down-regulates in OS tissues and cells, OS patients with high level of miR-429 had better prognosis

**Figure 1:**
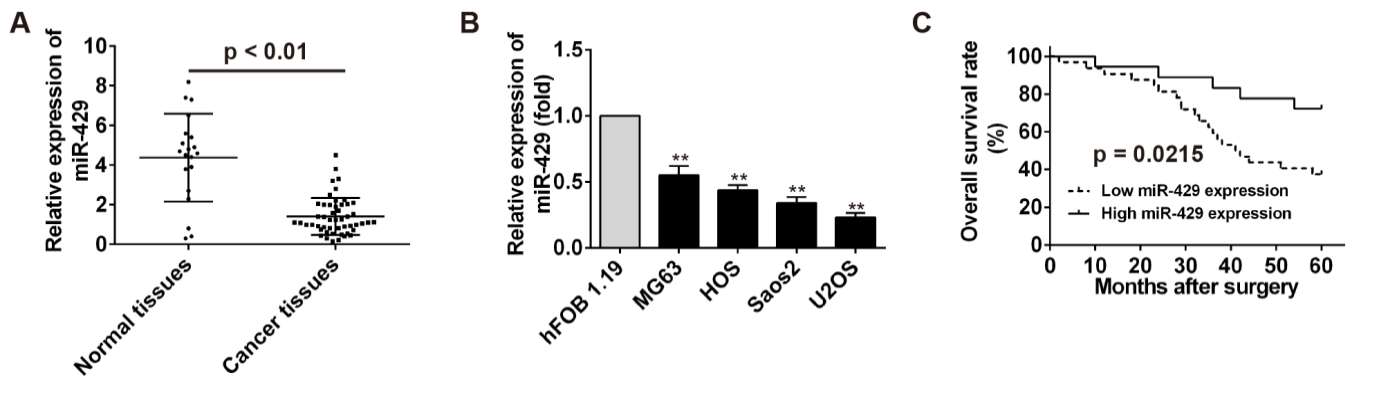
The clinicopathological features of miR-429 expression in osteosarcoma. A: The detection of miR-429 expression in osteosarcoma tissues using real time PCR. B: The detection of miR-429 expression in osteosarcoma cell lines using real time PCR, ** *P* < 0.01 vs hFOB 1.19 cells. C: The survival curves based on miR-429 relative expression and overall survival was plotted using Kaplan-Meier method.

MiR-429 level in OS tissues and OS cells was detected by real time PCR and compared with that in normal tissues and hFOB cells. The results showed that miR-429 level in OS tissues and OS cells were significantly higher than that in normal tissues and hFOB cells (*P* < 0.01) (Figure 1A, B(Fig. 1)). Furthermore, patients with osteosarcoma were divided into two groups, high miR-429 expression group and low miR-429 expression group, to analyze the relationship between overall survival of patients with osteosarcoma and miR-429 expression using Kaplan-Meier survival analysis. High level of miR-429 was associated with high overall survival rate for patients with osteosarcoma (*P* = 0.0215) (Figure 1C(Fig. 1)). These results indicate that miR-429 was down-regulated in OS tissues and cells and related to the prognosis of OS patients.

### Overexpression of miR-429 suppresses the invasion, metastasis, proliferation and promotes apoptosis of OS cells in vitro

**Figure 2:**
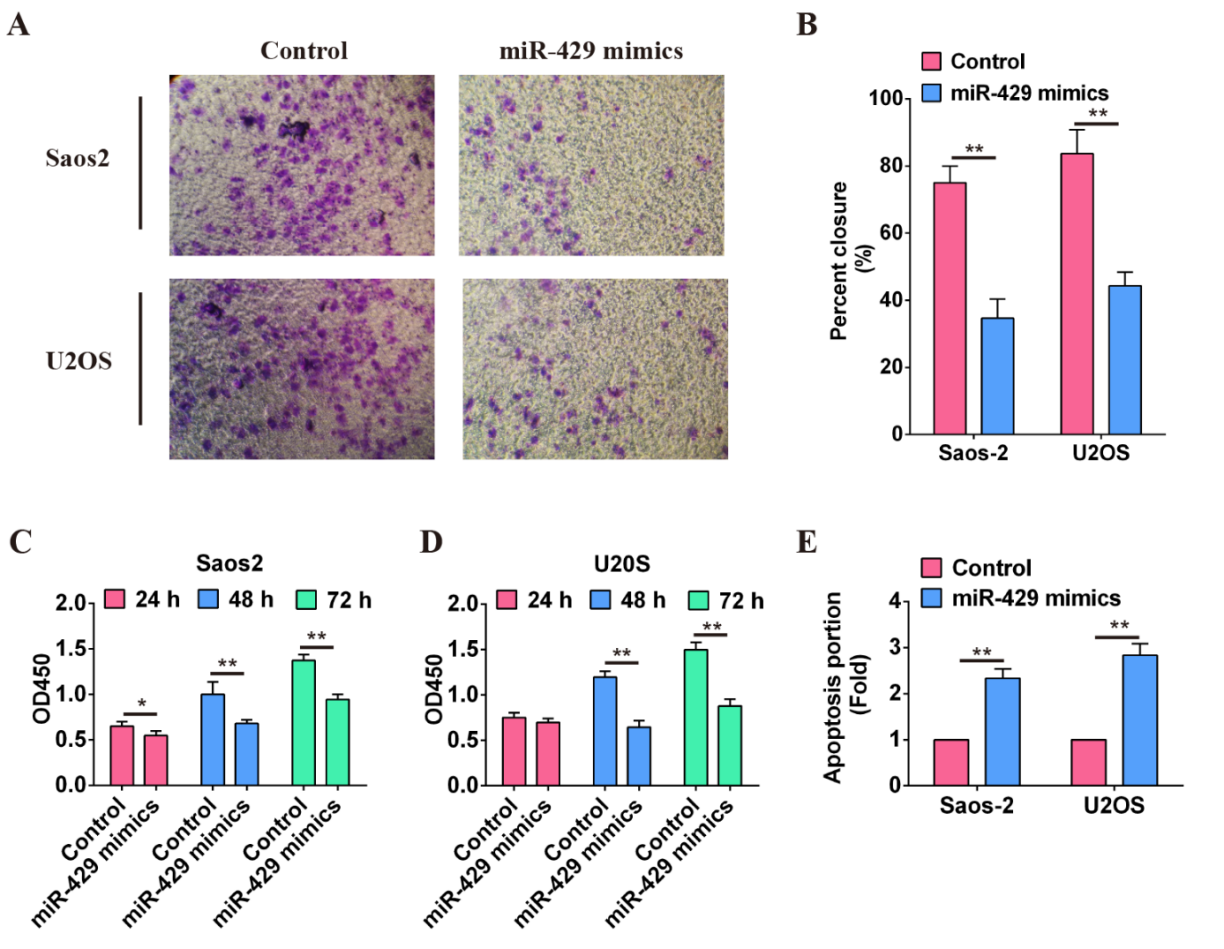
Function verification of miR-429 in osteosarcoma cells. A: Invasive ability of osteosarcoma cells with high level of miR-429 was significantly attenuated compared with that of cells in control group. B: Migration ability of osteosarcoma cells with high level of miR-429 was evaluated using wound-healing assay, ** *P* < 0.01. C: Proliferation of osteosarcoma cells with high level of miR-429 was evaluated within 72 h using CCK-8 assay, * *P* < 0.01, ** *P* < 0.01. D: Cell apoptosis of osteosarcoma cells was induced by miR-429, ** *P* < 0.01.

The functions of miR-429 in OS cells were evaluated on the basis of transwell invasion assay, wound-healing assay, CCK-8 proliferation assay and cellular apoptosis assay. The results of transwell invasion assay showed that invasive ability of OS cells (Saos2, U2OS) transfected with miR-429 mimics was decreased obviously than that of cells transfected with mimics NC (Figure 2A(Fig. 2)). This was consistent with the results of wound-healing assay (*P* < 0.01) (Figure 2B(Fig. 2)). The proliferative capability of OS cells was detected at 24 h, 48 h and 72 h after transfection with miR-429 mimics or mimics NC, respectively. The results showed that the proliferative capability of OS cells transfected with miR-429 mimics was attenuated compared with that of cells transfected with mimics NC, and the difference was more apparent at 48 h and 72 h (*P* < 0.01) (Figure 2C and 2D(Fig. 2)). Apoptosis portion of OS cells transfected with miR-429 was significantly lower than that of cells transfected with mimics NC (*P* < 0.01) (Figure 2E(Fig. 2)). These results suggest that high level of miR-429 may function as a suppressor miR in OS cells and inhibits the invasion, metastasis, proliferation of OS cells but induces OS cells' apoptosis.

### Overexpression of miR-429 up-regulates E-cadherin protein expression and down-regulates the protein expression of Vimentin, N-cadherin and Snail in OS cells 

**Figure 3:**
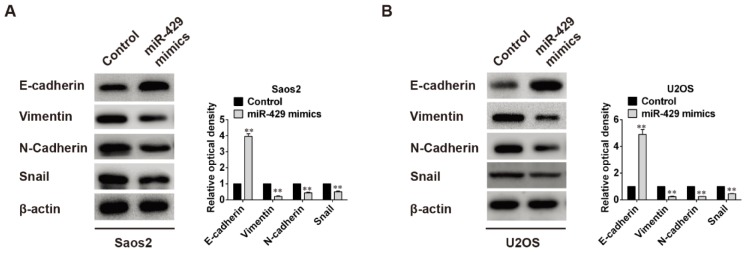
Effects of miR-429 overexpression on EMT related proteins expression. A: High level of miR-429 increased epithelial marker E-cadherin expression but decreased mesenchymal markers (Vimentin, N-Cadherin, Snail) expression in Saos2 cells, ** *P* < 0.01 vs control group. B: Same changes in U2OS cells as in Saos2 cells, ** *P* < 0.01 vs control group.

To evaluate the effects of miR-429 on epithelial-mesenchymal transition (EMT), proteins related to EMT, such as E-cadherin, Vimentin, N-cadherin and Snail, were detected using Western blot and the relative optical density was analyzed too. Whether in Saos2 cells or U2OS cells, the protein level of E-cadherin was raised obviously whereas protein levels of Vimentin, N-cadherin and Snail were all dramatically declined when cells were transfected with miR-429 mimics (*P* < 0.01) (Figure 3(Fig. 3)), indicating that high level of miR-429 may depress EMT of OS cells. 

### ZEB1 is the target gene of miR-429 and ZEB1 mRNA expression is inversely related to miR-429 expression in OS tissues

**Figure 4:**
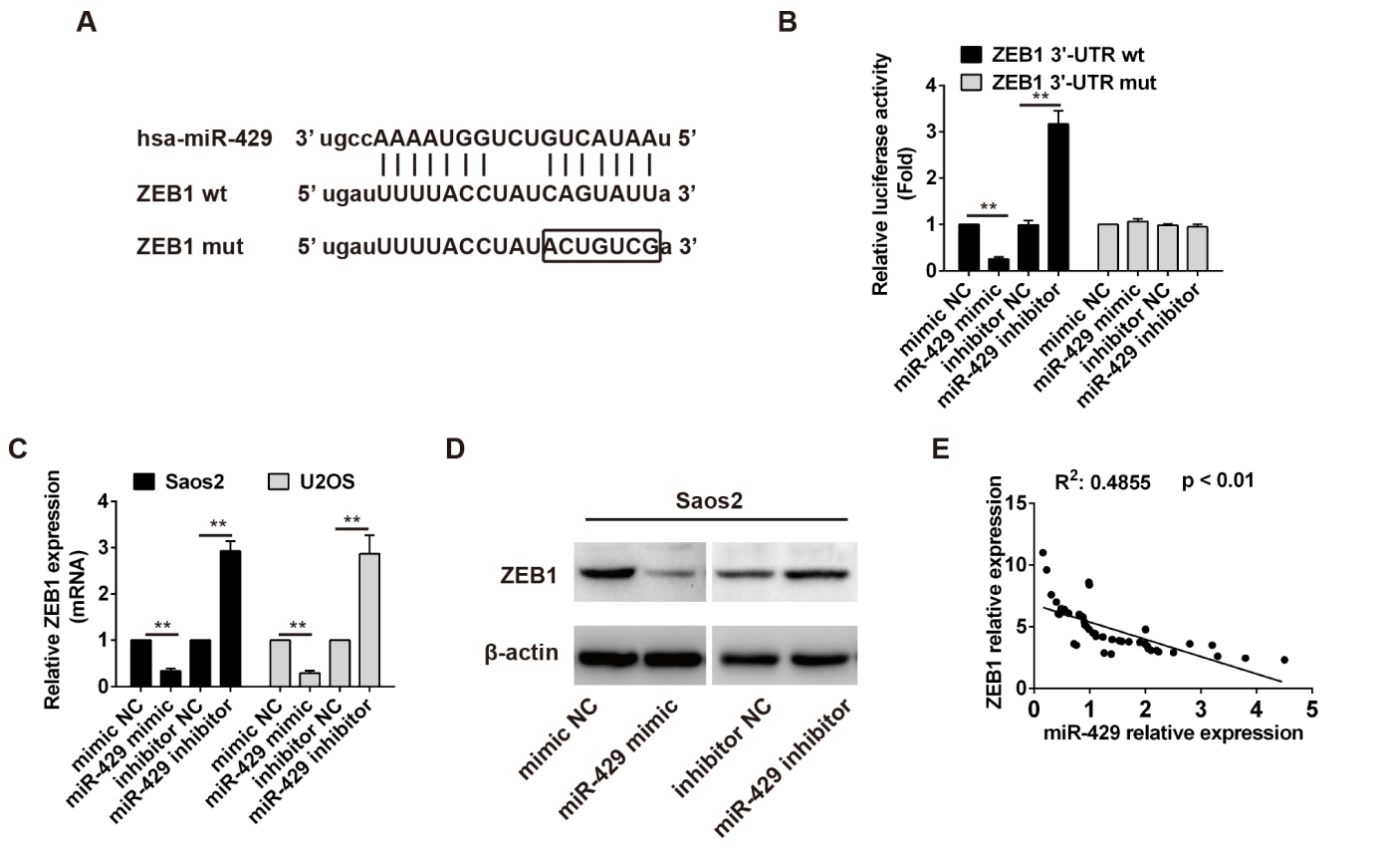
Prediction and verification of target gene of miR-429 in osteosarcoma. A: The binding site prediction between miR-429 and 3'-UTR region of ZEB1 mRNA. B: Relative luciferase activity detection, ** *P* < 0.01. C: ZEB1 mRNA expression in osteosarcoma cells with different treatments, ZEB1 mRNA level was normalized to GAPDH, ** *P* < 0.01. D: ZEB1 protein expression in osteosarcoma cells with different treatments. E: Correlation analysis between ZEB1 relative expression and miR-429 relative expression using Pearson's correlation.

pmirGLO-ZEB1 3'-UTR wt and pmirGLO-ZEB1 3'-UTR mut vectors were constructed according to the binding site between miR-429 and ZEB1 mRNA (Figure 4A(Fig. 4)). HEK 293T cells were cultured and cotransfected with pmirGLO-ZEB1 3'-UTR wt or pmirGLO-ZEB1 3'-UTR mut and mimics NC or miR-429 mimics or inhibitor NC or miR-429 inhibitor. The relative luciferase activity of reporter with ZEB1 3'-UTR wt was significantly decreased in cells with miR-429 mimics compared with that in cells with mimics (*P* < 0.01), and that in cells with miR-429 inhibitor was obviously increased than that in cells with inhibitor NC (*P* < 0.01). But the relative luciferase activity of reporter with ZEB1 3'-UTR mut had no obvious change among different groups (Figure 4B(Fig. 4)). Similarly, the relative expression of ZEB1 mRNA and ZEB1 protein in OS cells transfected with mimics NC or miR-429 inhibitor were both significantly higher than that in OS cells transfected with miR-429 mimics or inhibitor NC (*P* < 0.01) (Figure 4C, D(Fig. 4)). The correlation analysis showed that the relative expression of ZEB1 mRNA was inversely related to the relative expression of miR-429 in OS tissues (R^2^: 0.4855, *P* < 0.01) (Figure 4E(Fig. 4)). Taken together, these results indicate that ZEB1 is a direct target of miR-429 and miR-429 can down-regulate the expression of ZEB1 in OS.

## Discussion

miR-429 functions as a tumor suppressor and miR-429 level is determined to be decreased in many types of cancer (Zhang et al., 2016[27]). Plenty of miRNAs have been reported to function as oncogenes or tumor suppressors in OS, like miR-214, miR-183, miR-27a, miR-133b, Mir-494, and so on (Ram Kumar et al., 2016[18]; Zhi et al., 2016[29]), but there are scarcely any researches about mechanism and function of miR-429 in OS (Liu et al., 2014[13]). So in this study, the miR-429 level in OS tissues and OS cell lines was firstly detected, and miR-429 was both down-regulated in OS tissues and OS cell lines. Furthermore, overall survival analysis suggests that high level of miR-429 is associated with favorable prognosis. Taken together, miR-429 may function as a tumor suppressor in OS.

Then analyses of OS cells invasion, metastasis, viability and apoptosis indicate that up-regulation of miR-429 can effectively suppress the invasion, migration and growth abilities of OS cells, but facilitates the apoptosis of OS cells *in vitro*. This consists with the effects of miR-429 in esophageal carcinoma, hepatitis B virus-related hepatocellular carcinoma, hepatocellular carcinoma (Zhang et al., 2015[28]). In addition, high level of miR-429 promoted E-cadherin protein expression but inhibited the expression of Vimentin, N-Cadherin and Snail proteins in OS cells *in vitro*. E-cadherin, an epithelial marker, is a calcium-dependent cell-cell adhesion glycoprotein, down-regulation of E-cadherin will enhance the cellular motility leading cancer cells to the mesenchymal state and cancer progression and metastasis (Polyak and Weinberg, 2009[17]). On the contrary, N-Cadherin, another calcium-dependent cell adhesion molecule, is normally found in mesenchymal cells and cancer cells to play a role in migration (Ramis-Conde et al., 2009[19]). Vimentin is the major cytoskeletal component of mesenchymal cells, also is a marker of cancer cells undergoing an EMT (Leader et al., 1987[10]). Snail is a family of zinc finger transcriptional repressor, can bind to E-cadherin and suppress the expression of E-cadherin inducing EMT in cancer cells (Kumarswamy et al., 2012[8]). EMT is a process in which cells lose intercellular adhesion and obtain migrartory and invasive capabilities. So EMT can enable the metastasis and invasion of cancer cells and is essential for cancer progression and metastasis (Chaffer and Weinberg, 2011[2]). Thus miR-429 may play an inhibitory role in multiple types of cancer via suppressing cancer cells EMT, metastasis, invasion, proliferation and promoting cells apoptosis.

At last, dual luciferase assay and transfection assay showed ZEB1 was a direct target of miR-429 and miR-429 could down-regulate the ZEB1 expression both in mRNA level and protein level in OS cells. Furthermore, there was a negative correlation between miR-429 expression and ZEB1 mRNA expression in OS tissues. ZEB1 is a zinc finger class of transcription factor, which is also an EMT inducer and aberrantly expressed in multiple cancers (Peinado et al., 2007[16]). Therefore ZEB1 may play a similar role in OS tissues and its expression and functions may be inhibited by tumor suppressor miR-429.

In summary, miR-429 functions as a tumor suppressor in OS and is down-regulated in OS. Overexpression of miR-429 not only inhibits the EMT, metastasis, invasion and proliferation of OS cells, but also facilitates OS cells apoptosis, thus high level of miR-429 is associated with the favorable prognosis of OS. Furthermore, miR-429 can directly down-regulate the EMT inducer ZEB1 expression, so tumor suppressor miR-429 may repress the progression and metastasis of OS at least partially through inhibiting ZEB1 expression.

## Acknowledgements

This work was supported by General Project of Chongqing Basic Science and Frontier Technology Research (Grant No.: cstc2014jcyjA10017) and Science and Technology Research Project of Chongqing Education Commission (Grant No.: KJ1702023).

## Conflict of interest

The authors declare no conflict of interest.
